# Explainable Artificial Intelligence Helps in Understanding the Effect of Fibronectin on Survival of Sepsis

**DOI:** 10.3390/cells11152433

**Published:** 2022-08-05

**Authors:** Anna Lemańska-Perek, Dorota Krzyżanowska-Gołąb, Katarzyna Kobylińska, Przemysław Biecek, Tomasz Skalec, Maciej Tyszko, Waldemar Gozdzik, Barbara Adamik

**Affiliations:** 1 Department of Chemistry and Immunochemistry, Wroclaw Medical University, M. Sklodowskiej-Curie 48/50, 50-369 Wroclaw, Poland; 2 Faculty of Mathematics, Informatics and Mechanics, University of Warsaw, Banacha 2, 02-097 Warsaw, Poland; 3 Faculty of Mathematics and Information Science, Warsaw University of Technology, 00-662 Warszawa, Poland; 4 Clinical Department of Anesthesiology and Intensive Therapy, Wroclaw Medical University, Borowska 213, 50-556 Wroclaw, Poland

**Keywords:** sepsis, fibronectin, biomarkers, survival prediction, artificial intelligence models

## Abstract

Fibronectin (FN) plays an essential role in the host’s response to infection. In previous studies, a significant decrease in the FN level was observed in sepsis; however, it has not been clearly elucidated how this parameter affects the patient’s survival. To better understand the relationship between FN and survival, we utilized innovative approaches from the field of explainable machine learning, including local explanations (Break Down, Shapley Additive Values, Ceteris Paribus), to understand the contribution of FN to predicting individual patient survival. The methodology provides new opportunities to personalize informative predictions for patients. The results showed that the most important indicators for predicting survival in sepsis were INR, FN, age, and the APACHE II score. ROC curve analysis showed that the model’s successful classification rate was 0.92, its sensitivity was 0.92, its positive predictive value was 0.76, and its accuracy was 0.79. To illustrate these possibilities, we have developed and shared a web-based risk calculator for exploring individual patient risk. The web application can be continuously updated with new data in order to further improve the model.

## 1. Introduction

Sepsis is a life-threatening condition caused by the body’s unbalanced response to an infection; it can rapidly lead to organ failure and death. It is the primary cause of death in intensive care units (ICUs), and the prognosis of patients with sepsis is often difficult. The severity of a patient’s condition on admission to the ICU can be determined using clinical scales such as the APACHE II score (Acute Physiology and Chronic Health Evaluation II), and the NEWS (National Early Warning Score), and the degree of organ dysfunction and outcome can be assessed daily by the SOFA score (Sequential Organ Failure Assessment), the SAPS (Simplified Acute Physiology) score, and the SSS (Sepsis Severity Score) [1,2]. Predicting mortality risk in ICU patients has numerous applications. It is practical for planning the allocation of resources and evaluating the performance of ICU wards. Mortality risk assessment is also used in clinical trials to characterize and compare patient groups, and is also an important part of quality assessment; risk-adjusted mortality is the most commonly used indicator of the quality of ICU care [3].

Accurately identifying patients with sepsis who are more likely to die and who can benefit most from additional monitoring or treatment remains a challenge. The additional use of biomarkers to help identify these patients is an attractive solution [4]. However, due to the heterogeneity and complex pathophysiology of sepsis, a single biomarker often provides insufficient information and cannot be reliably qualified as a predictor of outcome in patients with sepsis. Artificial intelligence prediction models, which have been shown to be useful for diagnosing and prognostication in other fields of medicine [5,6,7,8,9], could potentially add much value to these areas for sepsis patients. Previous studies have demonstrated the effectiveness of machine learning algorithms in detecting sepsis early in general patient populations, showing an improvement in the early identification of at-risk patients [10,11]. In another study, the use of a machine learning algorithm was associated with improved sepsis patient outcomes; statistically significant differences for sepsis patients were found for the length of stay and in-hospital mortality [12].

Our previous study showed that fibronectin (FN) levels are associated with clinical indices of sepsis severity and could be used as a predictor of outcome [13]. Fibronectin, a key component of the extracellular matrix (ECM), is an adhesive dimeric glycoprotein with variable molecular conformations and splice variants. It is involved in several processes, including vascular development, wound healing, and ECM remodeling [14]. There are two defined types of FN: soluble plasma fibronectin (pFN) synthesized by hepatocytes, and insoluble cellular fibronectin (cFN) synthesized locally by different cell types, which accumulates in tissues as a component of the ECM [14,15]. Because of alternating splicing of the FN gene, the cellular form of FN contains two extra domains, A and B (EDA and the EDB isoform), which are absent or present in trace amounts in the blood of a healthy person, but their level rapidly increases in various pathological conditions, including sepsis [13,16,17]. The plasma form of FN is involved in haemostasis as an important component of blood clots, and, through its interaction with fibrin, plays a significant role in the coagulation cascade [18]. Cellular FN promotes inflammatory processes by activating the toll-like-receptor-4 signaling pathway. Furthermore, cellular FN is a ligand for integrin-91 that is expressed on various inflammatory cells, such as neutrophils and macrophages [19]. Fibronectin is a target for many bacterial proteins, and as part of a three-component bridge (fibronectin–integrin–fibronectin binding proteins), it contributes to the bacterial colonization of endothelial and epithelial cells [20,21].

In this paper, we demonstrate the efficacy of a machine learning model based on a random forest algorithm, developed to predict the probability of patient survival of sepsis on admission to the ICU. In addition to the commonly used indicators of the clinical condition of sepsis patients, the model included the fibronectin concentration recorded on the day of admission to the ICU. Moreover, we used Explainable Artificial Intelligence (XAI) techniques to better understand the model predictions. XAI techniques are becoming widely used in medicine [22,23]. In our study, we present two local level explanation techniques that assist in understanding the model prediction for a specific patient; these tools identify which features are the most important for a patient and indicate the change that occurs in the prediction with a change in the value of a variable. Such insight is much more interesting than averaged model behavior, both for an individual patient and for the physician. Therefore, these approaches can be considered personalized medicine. An online application of the model was developed, which presents the survival prognosis for both an individual patient and the entire data set in the model. This is a preliminary study to further develop the machine learning model, and data on patients with sepsis will be systematically added.

## 2. Materials and Methods

### 2.1. Study Group

This observational retrospective study included patients with sepsis/septic shock admitted to the Intensive Care Unit (ICU) in a tertiary-care university hospital. The analysis of plasma and cellular forms of fibronectin was conducted at the Department of Chemistry and Immunochemistry of Wroclaw Medical University. The survival prediction model and the online application of the machine learning model were created at the Faculty of Mathematics, Informatics and Mechanics, University of Warsaw. The study was conducted according to the guidelines of the Declaration of Helsinki and approved by the Institutional Review Board Bioethics Committee of the Medical University in Wroclaw (No. 637/2014), and informed consent was obtained from the patients or their representatives.

Inclusion criteria were: age >18 years and a diagnosis of sepsis/septic shock, according to the Sepsis-3 definition on admission to the intensive care unit [2].

Exclusion criteria were: previously treated at the ICU, pregnancy, terminal illness with no chance for meaningful recovery or expected ICU length of stay of 24 h or less.

All patients admitted to the ICU who met the inclusion criteria were included in the study. All patients in the study received standard treatment for septic shock according to the Surviving Sepsis Campaign guidelines [24]. Demographic, laboratory and clinical data were collected from patient medical records.

The severity of the clinical status of the patient on admission to the ICU was determined using the APACHE II (Acute Physiology and Chronic Health Evaluation II) score [25]. The score is made up of 12 physiological variables (the fraction of inspired oxygen, partial pressure of oxygen, body temperature, mean arterial pressure, blood pH, heart rate, respiratory rate, serum sodium, serum potassium, serum creatinine, hematocrit, white blood cell count, and the Glasgow Coma Scale) and 2 disease-related variables (history of severe organ failure or immunocompromise and the type of ICU admission).

The degree of organ dysfunction of patients on admission to the ICU was assessed with a Sequential Organ Failure Assessment (SOFA) score. The score is used in the ICU for monitoring the severity of sepsis based on the status of six body systems: respiratory (PaO2/FiO2 index), cardiovascular (mean arterial pressure and the dose of vasopressors), hepatic (serum bilirubin level), coagulation (platelets level), renal (serum creatinine level/urine output), and neurological (Glasgow Coma Scale). Both the APACHE II and SOFA scores are routinely used tools for ICU patients. Demographic data and laboratory parameters, such as the white blood cell (WBC) count, C-reactive protein (CRP) level, procalcitonin (PCT) level, and coagulation parameters (d-dimers; international normalized ratio, INR) were also recorded.

### 2.2. Blood Sample Collection and Fibronectin Concentration Measurement

Blood samples (2.7 mL), anticoagulated with 3.2% sodium citrate, were collected from patients diagnosed with sepsis or septic shock on the day of admission to the ICU. Plasma was separated by centrifugation at 2000 rcf for 10 min, aliquoted, and stored at −70 °C for fibronectin measurements.

#### 2.2.1. Plasma FN Concentrations

Plasma FN concentrations were determined by an enzyme-linked immunosorbent assay (ELISA) using a well-defined domain-specific monoclonal antibody directed to the cell-binding domain of FN (FN30-8; M010 TaKaRa Shuzo Co. Ltd., Shiga, Japan), as described earlier [26]. Briefly, the monoclonal antibody directed to the cell-binding domain of FN (FN30-8 M010, diluted 1:10,000) was used as a coating agent in the wells of a microtiter plate (Nagle Nunc International, Naperville, IL, USA) to bind FN from the samples. The amount of FN bound by the monoclonal antibody was quantified using rabbit anti-FN polyclonal antibodies (Sigma Chemical Co., St Louis, MO, USA, diluted 1:5000) and secondary antibody peroxidase conjugated goat anti-rabbit immunoglobulins (Sigma Chemical Co., St Louis, MO, USA, diluted 1:30,000). The test was assayed by a colorimetric reaction using o-phenylenediamine dihydrochloride/H_2_O_2_ as the enzyme substrate. The results were expressed in absorbance units (AU). The samples were analyzed in two different dilutions, each in duplicate. The pFN concentration is given in milligrams per liter. A human plasma FN preparation (Sigma Chemical Co., St. Louis, MO, USA, from 1.5 to 50 ng/well) was used as a standard for determining the pFN-ELISA.

#### 2.2.2. EDA-FN Concentrations

The EDA-FN concentration was determined by ELISA using a domain-specific primary antibody (S-FN5, clone IST-9, Sirius Biotech S.r.l., Genoa, Italy) and a biotinylated secondary antibody (715-066-151, Jackson ImmunoResearch, Baltimore, MD, USA). The detection of EDA-FN in the plasma was based on the method described earlier [27]. Briefly, gelatin (0.5% in TBS/well overnight at 4 °C) was used as a coating agent on ELISA plates (Nalge Nunc International, Naperville, IL, USA). FN from the plasma samples was bound to the gelatin; next, an IST-9 monoclonal antibody (1.0 µg/mL, Sirius Biotech S.r.l., Genoa, Italy) was used to detect FN containing the EDA-FN domain. Biotinylated donkey anti-mouse antiserum (diluted 1:10,000, Dianova GmbH, Hamburg, Germany) was added in the next step, and finally, horseradish peroxidase (HRP)-labelled streptavidin (1 µg/mL, Dianova GmbH, Hamburg, Germany) was added. Incubation with the secondary antibody and peroxidase-conjugated Streptavidin was performed without access to light. The test was assayed by a colorimetric reaction using o-phenylenediamine dihydrochloride/H2O2 as the enzyme substrate. A cellular fibronectin from human foreskin fibroblasts (Sigma Chemical Co., St. Louis, MO, USA, from 1.5 to 50 ng/well) was used as a standard. The samples were analyzed in two different dilutions, each in duplicate. The EDA-FN concentrations are given in milligrams per liter. To determine non-specific binding, two controls were included in the tests: without the primary antibody and without the secondary antibody.

### 2.3. Statistical Methods

**Figure 1:**
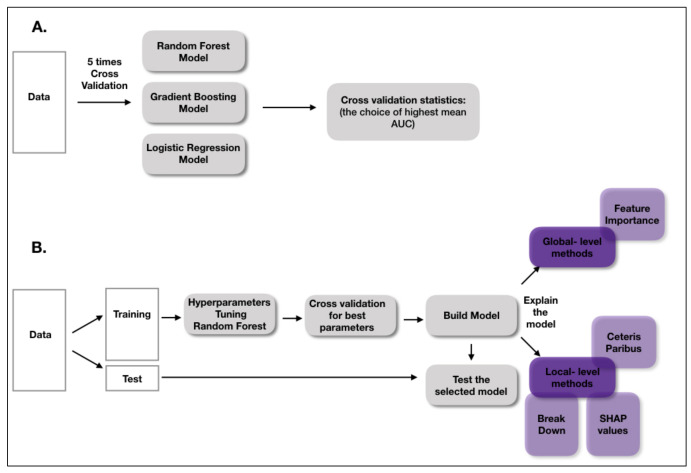
A workflow diagram. (**A**) This part presents how the best model was chosen. (**B**) The workflow of building and explaining the final model. Results are available at: https://stats4med.shinyapps.io/xai2shiny/ (accessed on 8 June 2022).

Continuous variables were summarized with three statistics: the median and the interquartile range between the 25th and 75th percentiles, while categorical variables were summarized as counts and fractions. A comparison of the continuous variables between two independent groups (Nonsurvivors vs. Survivors) was performed using the Mann–Whitney test. Categorical variables were analyzed with the chi-squared test, and contingency tables were used to analyze the frequency distribution of categorical variables. *p*-values less than 0.05 were regarded as significant. This study presents the effectiveness of an artificial intelligence model designed to predict the probability of survival in sepsis. Figure 1 shows a workflow diagram. Three types of models were developed: a logistic regression model and two complex, tree-based models: random forest and gradient boosting [28,29]. Logistic regression is our choice for a transparent model that is widely used in the medical domain. Random forest and gradient boosting, the so-called black box models, are very often more accurate, but at the cost of interpretability.In order to understand the machine learning model predictions, we used two types of Explainable Artificial Intelligence (XAI) methods: global-level methods and instance-level methods. Global level methods assist in understanding the overall model structure. We used the model-agnostic Feature Importance method, which has been previously described in detail by Fisher et al. [30]. Briefly, the Feature Importance method measures changes in model performance after the perturbation of a variable. The bigger the loss of model performance after perturbation of a selected variable, the more important that variable is. The method helps to determine which variables influence the final prediction the most, but also which variables are not important.

Moreover, three local-level methods, Break Down, Shapley Additive Explanations (SHAP values) and Ceteris Paribus, were applied to the model [31]. The local-level methods helped us to understand how a model behaved for a particular, selected patient. The Break Down method [32] and SHAP values [33] presented how the variables contributed to the final prediction. However, the decomposition of the model predictions into the attribution of each variable was computed in a different way: the value of the attribution depended on the order of the explanatory variables for the Break Down method, whereas the SHAP values averaged the value of variable attribution over all possible orderings. Ceteris Paribus profiles showed the dependencies between continuous variables and model predictions. The method presented how a prediction would change if the value of a single variable changed for a selected instance. All analysis was performed with R 3.6.1 [34] with mlr [35] and DALEX [36] packages.

We have created a GitHub repository with code needed to prepare the models and explain them: https://github.com/KasiaKobylinska/XAIForSepsis (accessed on 8 June 2022).

## 3. Results

A total of 127 consecutive patients who were treated for sepsis/septic shock between January 2018 and December 2019 were screened for inclusion/exclusion criteria. Of this number, 122 patients met the inclusion criteria and were included in the final analysis. Five patients were excluded due to incomplete data records. Sepsis/septic shock was diagnosed on the basis of Sepsis-3 diagnostic criteria [2]. All patients on admission had a clinical suspicion of infection diagnosed by the attending physician on the basis of the source of infection (clinical, radiological or microbiological). Body fluids were routinely collected for culture from each patient on admission to the ICU: 99 patients had microbiologically confirmed bacterial infections. Moreover, samples taken for culture on subsequent days also confirmed bacterial infections in the remaining patients with sepsis. The most frequent primary sources of sepsis were abdominal (45%) and pulmonary (34%) infections. The 28-day mortality was 44%, and patient characteristics are summarized in Table 1. The patients who survived were younger, had lower APACHEII and SOFA scores, and lower levels of pro-inflammatory markers such as procalcitonin, c-reactive protein, and leukocytes.

### 3.1. Fibronectin Concentrations

Both forms of FN, plasma (pFN) and cellular (EDA-FN), were measured on admission to the ICU. The median pFN concentration was 114.06 mg/L; in Nonsurvivors, the pFN values were significantly lower compared to the values recorded in Survivors (83.02 mg/L vs. 138.82 mg/L, *p* < 0.001). The median value of the EDA-FN concentration in Nonsurvivors was higher than the median value measured in Survivors, but there was no statistically significant difference between the groups (9.4 mg/L vs. 5.04 mg/L, *p* = 0.055).

### 3.2. Results of Modeling

**Figure 2:**
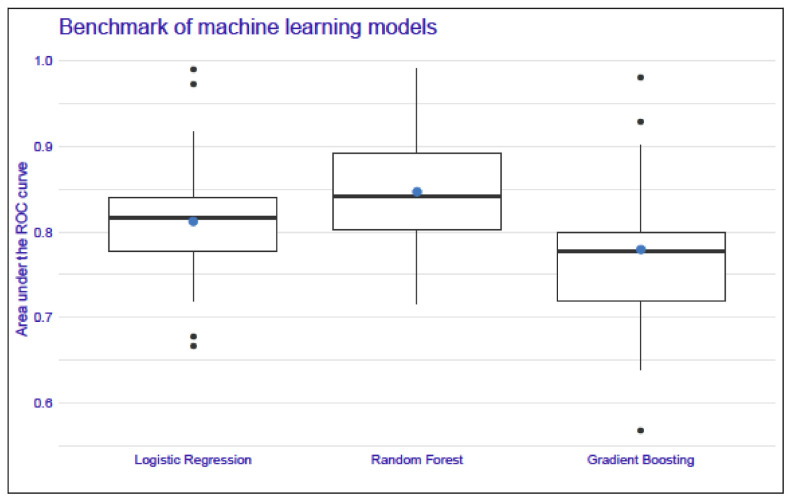
A comparison of the area-under-the-curve values of the logistic regression, random forest, and gradient boosting models. The mean test AUC was the highest for the random forest model, whereas the lowest mean test AUC was for the boosting model. The blue dot represents the mean and the bold midline represents the median of the AUC results, whereas the upper and lower limits of the boxes correspond to the third and first quartiles. Black dots represent outliers in the data. Each boxplot presents the results of a 5-fold cross-validation procedure repeated five times for a specific model.

We took into consideration three types of models: the logistic regression, random forest and gradient boosting models. After running the models, we also prepared a benchmark to compare the results with different test data sets. The benchmark consisted of dividing the input data set into test and training sets, preparing the model on the training data set and computing the area under the curve (AUC) on the test data set. The procedure was repeated five times. The proportion of the test to training patients in the sets was 1:2. The mean test AUC was 0.85 for the random forest model, 0.78 for the gradient boosting model, and 0.81 for the logistic regression model. The results of the test AUCs for the models are presented in Figure 2.

**Figure 3:**
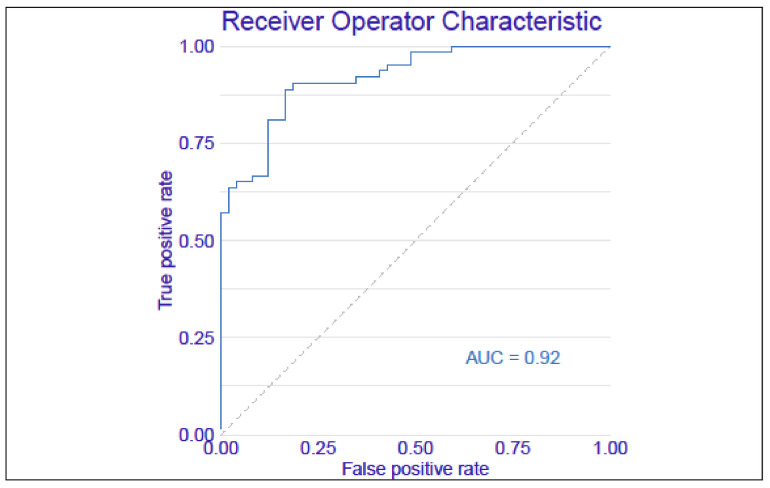
The ROC curve of the machine learning model for predicting the survival of sepsis. The following input variables were included in the model: pFN, the INR value, the APACHE II score, age, the SOFA score, the platelet count, the procalcitonin level, and the d-dimers level.

The best results were obtained for the random forest model, and this model is discussed further. The independent variables which were put into the model were selected based on the significance tests presented in Table 1 and based on the results obtained for fibronectin. Additionally, d-dimers were included in the analysis as a parameter indicative of fibrin degradation. In our previous study, we detected the presence of fibronectin-fibrin complexes in the plasma of sepsis patients; furthermore, the frequency of occurrence and the relative amount of fibronectin–fibrin complexes were higher in Nonsurvivors than in Survivors [13]. The machine learning model was developed with input features of the concentration of plasma fibronectin, the INR value, the SOFA score, the patient’s age, the APACHE II score, the procalcitonin level, the platelet count, and the level of d-dimers. A 10-time cross-validation was performed to optimize the random forest model parameters and unique overfitting. The mean AUC of the 10-time cross-validation computed for the test data sets was 0.82. The final model was prepared on the training data set. The ROC curve analysis of the random forest model showed that the rate of successfully classifying patients with the model was 0.92 (AUC computed on the whole data set) (Figure 3), with a sensitivity of 0.92 (recall), positive predictive value of 0.76 (precision), and accuracy of 0.79 obtained.

### 3.3. Global-Level Methods for Model Explanation

When making high-risk decisions, it is crucial to understand the structure of a model and the dependencies contained within. As it was difficult to interpret the black box model, we applied explanatory methods, such as Feature Importance, Break Down, SHAP values and Ceteris Paribus, which can help physicians interpret the results. The global-level perspective was applied in order to understand the model structure and the dependencies between the patient features and the model predictions. The permutational variable importance method indicated which variables were significant to the model response. The approach of this method was to compare the performance of the model with the performance after some variable permutations. The larger the loss functions, the more important the selected variable is.

### 3.4. Feature Importance with the Random Forest Model

**Figure 4:**
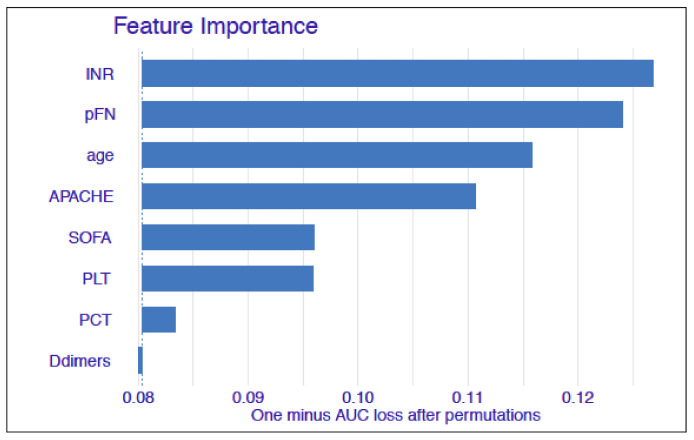
The Feature Importance plot showing the most significant variables for the model. The length of the bar indicates the loss in the AUC when a given variable was altered. The bigger the loss, the more important the variable is.

The importance of specific features for predicting the probability of patient survival is presented in Figure 4. The longer the variable’s bar, the more significant this feature was in the random forest model. According to the plot, the INR value and the concentration of plasma fibronectin were the most important variables for the random forest model. The APACHE II score and age were also important variables for the model. The model indicated that the SOFA score, platelet count, and procalcitonin level were less informative variables. The level of d-dimers did not seem to have an impact on the model predictions.

### 3.5. Local-Level Methods for Model Explanation

We also applied the local explaining methodology in order to support medical decision making for an individual patient. We used the Ceteris Paribus method, which presents the dependency between the possible model responses, if the change in one feature would have occurred for a selected instance. We used the Break Down method and the SHAP values, which separate the model prediction into each variable contribution for a selected instance. These methods were helpful in understanding which variables were the most important for a selected patient, and how they influenced the model’s result.

#### Example of Clinical Application of the Random Forest Prediction Model

The local methods presented in the article were applied to one selected patient, but could be extended to other patients thanks to the online application. The application created for predicting the survival of septic patients is based on the random forest model described above. The following input variables were included: plasma fibronectin, the INR value, the platelet count, the APACHE II score, age, the SOFA score, the procalcitonin level, and the level of d-dimers. The online application is available at https://stats4med.shinyapps.io/xai2shiny/ (accessed on 8 June 2022), and the analysis presented below is an example of the use of the model.

**Figure 5:**
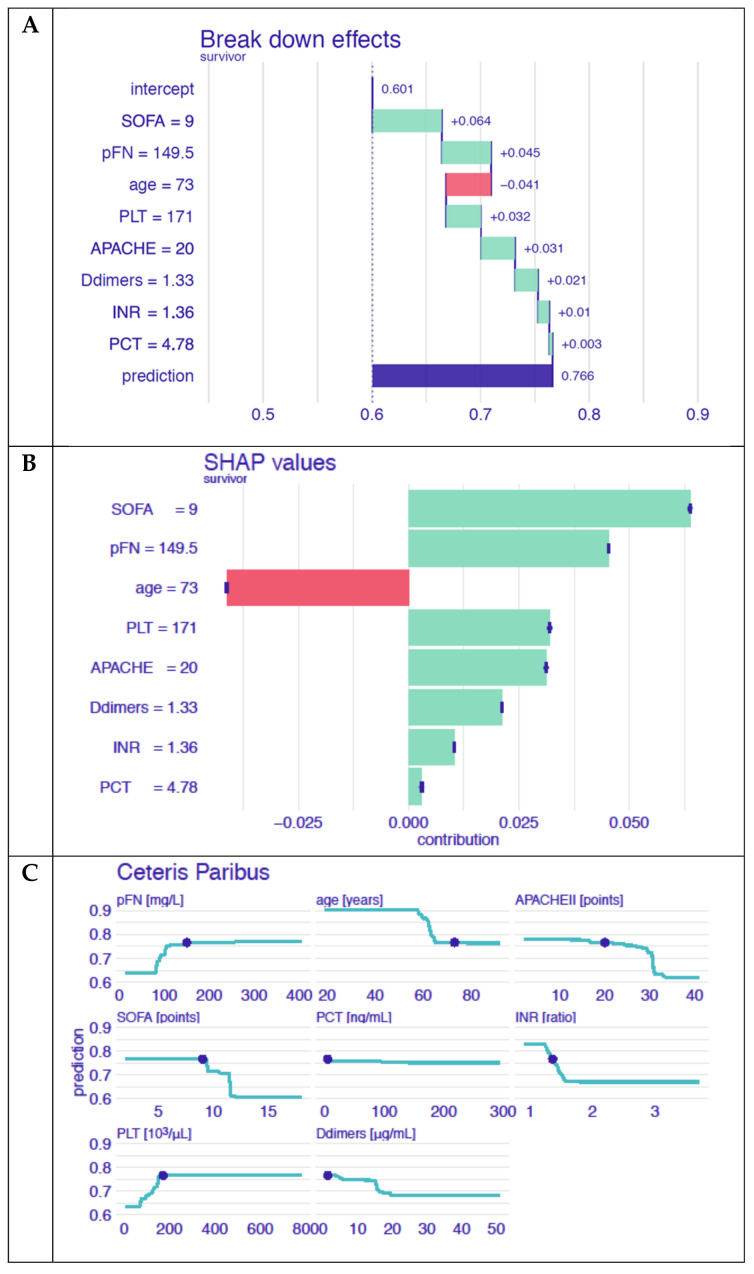
The survival prediction model for a selected patient. (**A**) The impact of the values of particular features on the survival prediction based on the Break Down method: an example based on data collected on admission to the ICU. (**B**) The impact of the values of particular features on the survival prediction based on SHAP values: an example based on data collected on admission to the ICU (**C**). The calculation of survival when one feature changed: an example based on data collected on admission to the ICU.

Analysis: The patient was admitted to the Intensive Care Unit directly from the operating theatre after relaparotomy due to the perforation of the caecum and fecal peritonitis. On admission, the patient was diagnosed with septic shock and given catecholamines and cordarone in order to stabilize the cardiovascular system. Due to respiratory failure, the patient was mechanically ventilated with high pressure and oxygen support. Empirical antibiotic therapy was implemented, and parenteral nutrition was introduced. Due to persistent oliguria, hemodiafiltration was implemented in citrate anticoagulation. The patient’s clinical status was assessed with the APACHE II score (20 pts.) and the SOFA score (9 pts.). The other parameters used in the predictive model were as follows: plasma fibronectin level 149.51 mg/L, procalcitonin 4.68 ng/L, d-dimers 1.33 mg/L, and INR 1.36. According to the presented random forest model, the prediction of 28-day survival calculated for this patient on admission to the ICU was 0.764, which was higher than the average model prediction. Based on the Break Down method, the most important variable in the model that increased the accuracy of the prediction was the SOFA score, with the contribution + 0.064, and the level of pFN, with the contribution + 0.45 (Figure 5A). Other variables were of less importance, and the only variable that had a negative impact on the prediction was age. Very similar results were obtained using the SHAP values (Figure 5B). Moreover, when taking into account the Ceteris Paribus profiles, we can see that a loss in the pFN value or even a small increase in the SOFA level resulted in a worse prediction for that patient (Figure 5C). According to the hospital documentation, the patient was alive on day 28 of treatment.

## 4. Discussion

In this study, we used a machine learning model with input features, including fibronectin and a new potential sepsis biomarker, in conjunction with routinely measured vital indices, to predict the survival of patients diagnosed with sepsis on admission to the ICU. Three types of models were developed: logistic regression, random forest and gradient boosting; based on the test and train AUC values, the random forest was chosen as the final model. Our results showed that the most important indicators for predicting survival were the INR and the level of plasma fibronectin, followed by age and the APACHE II score.

On the basis of the data from the analyzed cohort, an online application was developed for predicting the survival of individual patients.

Sepsis is a major cause of death in the ICU, and much research has been done to test models for the early prediction of sepsis diagnosis and outcome [37,38,39]. In addition to the clinical scores routinely used to assess the clinical status of patients with sepsis, and basic demographic parameters such as age, gender, and race, biomarkers have been shown to be useful in the early diagnosis of sepsis and prognosis of outcome. Numerous studies indicate that protein biomarkers can be valuable parameters in such models, increasing the accuracy of diagnosis and prognosis [5,40,41,42].

Fibronectin has previously been proposed as a biomarker of sepsis: it was observed that low concentrations of FN in the plasma of patients who were suspected to have sepsis were consistent with the final diagnosis of sepsis and a positive blood culture result [43]. Decreased plasma levels of FN have also been observed in acute inflammation, surgical trauma and disseminated intravascular coagulation [44,45]. Mamani et al. proposed FN as a diagnostic marker of sepsis; however, the ROC curve analysis and a comparison of the AUC values for FN and CRP showed that the diagnostic value of CRP was significantly higher [46]. In our previous work, we showed that the plasma FN concentration measured in septic patients was significantly lower than in healthy adult controls [13]. In addition, we found that plasma FN levels in Nonsurvivors were significantly lower than in Survivors, and particularly low levels of FN were measured in septic patients with DIC. All these observations suggest the potential usefulness of FN as a prognostic marker in sepsis. However, previous attempts to search for prognostic biomarkers in patients with systemic inflammatory response syndrome (SIRS) have not shown the usefulness of FN in both univariate and multivariate logistic regression analysis [44]. FN is a multifunctional protein; therefore, changes in the protein level may be the result of many different processes or effects. Fibronectin is a key component of a fibrin clot; decreases in the FN concentration in sepsis may result from the consumption of coagulation factors caused by intensified coagulation processes. We previously observed the formation of fibrin–fibronectin complexes in the plasma of patients with sepsis [13]. Moreover, FN is involved in haemostasis, rapidly depositing on the wall of the damaged vessel and promoting platelet aggregation through pFN–fibrin complexes, but interestingly, in the absence of fibrin, fibronectin inhibited this process [18]. One of the reasons for the decrease in the level of FN concentration in the plasma of sepsis patients is the cleavage of FN in pathological conditions linked with extracellular matrix remodeling and the release of FN fragments with pro-inflammatory and degradative properties. Our previous study confirmed that FN cleavage resulted in the presence of FN fragments with a mass mainly from 150 kDa to 70 kDa [13].

In addition to the plasma FN, we also measured the cellular form of FN, and found significantly higher levels of this FN form in septic patients than in healthy people [13]. This is consistent with previous reports of a rapid increase in cellular FN levels in pathological conditions, including sepsis [13,47,48]. Unlike plasma FN, we found no significant difference between the levels of cellular FN recorded in Nonsurvivors and Survivors. This is not surprising, given that these two FN isoforms are produced and function independently [14]. Our last study showed that the level of the cellular form of FN is related to treatment outcome and is significantly higher in COVID-19 Nonsurvivors than in Survivors. Additionally, EDA-FN levels correlated with APACHE II and SOFA scores [49].

In this study, we initially applied input features to the machine learning model of the concentrations of both forms of fibronectin (plasma FN and cellular FN). The plasma FN was the most important feature in the model, while the cellular form of FN was not significant for the model and was excluded from the final analysis. The ROC curve analysis of our prediction model presented in Figure 3 showed that the rate of successful classification by the model was as high as 0.92 with a sensitivity of 0.92, and had a positive predictive value of 0.76 (precision), and an accuracy of 0.79. As shown in Figure 4, the most important variable in the model was the INR value, followed by the plasma FN concentration, age, and the APACHE II score. Additionally, the SOFA score, platelet count, and the levels of procalcitonin and d-dimers were entered into the model as less informative variables.

On the basis of the input data from the analyzed cohort, we have created a web application for predicting the survival of individual patients: https://stats4med.shinyapps.io/xai2shiny/ (accessed on 8 June 2022). This is a preliminary design, limited by the small amount of data, although it will be easy to extend it in the future. The model will learn and develop as the database is expanded with parameters from the next cohort, and the application could serve as a survival prediction tool for outcome prognosis in sepsis.

Our study has several limitations. The number of cases was small, and we view the created model as a preliminary one that will be developed. All the analyzed patients had severe infections of bacterial origin. To continue the project, we intend to extend the database to include cases of bacterial and viral sepsis. There are limitations to the model itself: the analysis was prepared on a small data set, so the machine learning model could have been overfitted. The next study should include an extended database with more observations. Then, future studies may address the interpretability of even more sophisticated artificial intelligence tasks.

## 5. Conclusions

To better understand the relationship between FN and survival, we utilized innovative approaches from the field of explainable machine learning, including local explanations, to understand the contribution of FN in predicting survival at the level of a single patient. The methodology provides new opportunities to personalize informative predictions for patients. To illustrate these possibilities, we have developed and shared a web-based risk calculator allowing exploration of individual patient risk. With the random forest model, we showed that the concentration of fibronectin, in combination with routinely assessed parameters, could be useful as a marker for predicting survival in sepsis. An online application developed for predicting survival for individual patients can be continuously expanded with new data.

## Figures and Tables

**Table 1:** Patient characteristics at baseline.

Parameter	All	Nonsurvivors	Survivors	*p*
	*n* = 122	*n* = 54	*n* = 68	
Age (years)	68.0	71.0	64.0	0.001
	(60.0–77.0)	(65.0–79.0)	(56.0–74.0)	
Female/male (*n*)	58/64	27/27	31/37	0.627
APACHE II score	24.0	28.0	20.0	<0.001
(points)	(17.0–29.0)	(22.0–33.0)	(15.0–26.0)	
SOFA score	10.0	11.5	9.0	<0.001
(points)	(8.0–13.0)	(10.0–15.0)	(7.0–11.0)	
Procalcitonin	8.38	14.57	4.47	<0.001
(ng/mL)	(1.76–30.4)	(3.90–34.20)	(0.80–15.47)	
C-reactive protein	192.3	197.3	186.7	0.726
(mg/L)	(112.6–302.5)	(123.0–307.9)	(100.4–302.5)	
INR	1.34	1.49	1.20	<0.001
	(1.16–1.60)	(1.32–1.80)	(1.12–1.43)	
Platelet count	182.5	138.5	209.5	0.001
(10^3^/μL)	(124.0–310.0)	(74.0–243.0)	(155.0–335.0)	
D-dimers	5.68	5.70	5.54	0.294
(μg/mL)	(3.64–12.59)	(3.97–15.59)	(3.37–11.47)	
ICU stay	9.5	5.5	12.5	<0.001
(days)	(4.0–18.0)	(3.0–12.0)	(6.5–29.5)	
Leukocytes	15.5	15.9	15.0	0.660
(10^3^/μL)	(11.0–22.5)	(9.7–22.5)	(11.2–22.9)	

APACHE II, Acute Physiology and Chronic Health Evaluation II; SOFA, Sequential Organ Failure Assessment; INR, international normalized ratio. Values are presented as median and quartiles; the *p*-value represents the difference between Nonsurvivors and Survivors.

## Data Availability

The data presented in the study are available on request from the corresponding author. The data have not been made publicly available, because they contain information that could compromise the privacy of the study participants.
